# Moving towards IoT Based Digital Communication: An Efficient Utilization of Power Spectrum Density for Smart Cities

**DOI:** 10.3390/s20102856

**Published:** 2020-05-18

**Authors:** Tariq Ali, Abdullah S. Alwadie, Abdul Rasheed Rizwan, Ahthasham Sajid, Muhammad Irfan, Muhammad Awais

**Affiliations:** 1 Electrical Engineering Department, College of Engineering, Najran University, Najran 61441, Saudi Arabia; asalwadie@nu.edu.sa (A.S.A.); miditta@nu.edu.sa (M.I.); 2 Department of Computer Science, Punjab Education System, Depaalpur 56180, Pakistan; liveneeo@gmail.com; 3 Department of Computer Science, Faculty of ICT, Balochistan University of Information Technology Engineering and Management Sciences, Quetta 87300, Pakistan; ahthasham.sajid@buitms.edu.pk; 4 School of Computing and Communications, Lancaster University, Bailrigg, Lancaster LA1 4YW, UK; m.awais11@lancaster.ac.uk

**Keywords:** IoT, digital communication, line codes, SES

## Abstract

The future of the Internet of Things (IoT) is interlinked with digital communication in smart cities. The digital signal power spectrum of smart IoT devices is greatly needed to provide communication support. The line codes play a significant role in data bit transmission in digital communication. The existing line-coding techniques are designed for traditional computing network technology and power spectrum density to translate data bits into a signal using various line code waveforms. The existing line-code techniques have multiple kinds of issues, such as the utilization of bandwidth, connection synchronization (CS), the direct current (DC) component, and power spectrum density (PSD). These highlighted issues are not adequate in IoT devices in smart cities due to their small size. However, there is a need to design an effective line-code method to deal with these issues in digital IoT-based communication for smart technologies, which enables smart services for smart cities. In this paper, the Shadow Encoding Scheme (SES) is proposed to transmit data bits efficiently by using a physical waveform in the smart cities’ ecosystem. SES provides a reliable transmission over the physical medium without using extra bandwidth and with ideal PSD. In it, the shadow copy of the repeating bitstream is forwarded, rather than repeating the actual stream again and again. The PSD is calculated with the help of mathematical equations to validate SES. MATLAB simulator is used to simulate SES and compared with other well-known digital line-code techniques. The bit error rate is also compared between SES and the chirp spread spectrum (CSS) for the specific data frames. The coordinates of the PSD graph are also shown in tabular form, which shows a vivid picture of the working conditions of various line codes.

## 1. Introduction

**Figure 1:**
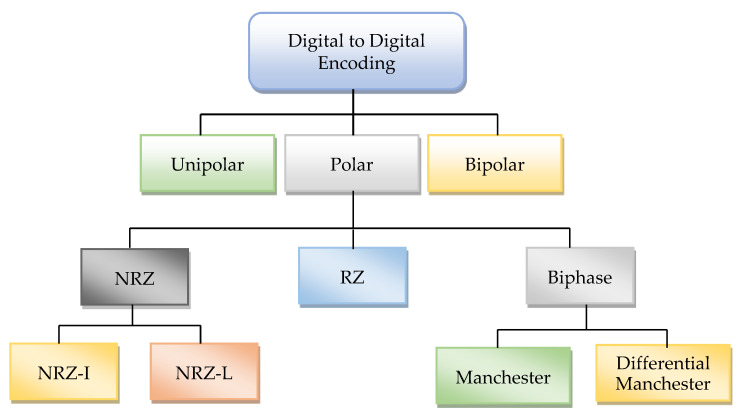
Digital encoding techniques.

The Internet of Things (IoT) is an emerging field in data communication. IoT devices operate with a very low power spectrum density due to their small size. The application of IoT in smart cities needs small devices to communicate with each other. The stability of IoT networks is highly dependent on “quality of service” (QoS) because, in the current situation, the bandwidth requirement for voice and video services is the primary concern for internet communication. One way to maintain the stability of service quality is to use a modulation technique, which authenticates the regulation of the signal pattern that depends on the signal-to-noise ratio (SRN) condition. Lower-order modulation can be used to maintain stability in case the channel condition is not good. Many digital conversion techniques have been proposed in the era of data communication to modulate the signal into waveform. These techniques are based on different signal modulation patterns. These techniques have their pros and cons for digital transmission. Some of them perform better against bandwidth utilization and rest for the power spectrum density (PSD) and direct current (DC) components. The signal format minimizes the effect of noise during message transmission. The choice of specific line code is based on its nature of the signal format [1,2]. The most valuable property of line code is PSD and the usage of bandwidth during signal transmission. The PSD shows the distortion of a signal in response to frequency. The ideal frequency of a signal ensures that the adopted line code does not have a DC component issue [3,4]. The primary purpose of a line code to transmit signals from one location to others should be less effect of noise and inter-symbol interference (ISI). The solution to ISI is also to increase bandwidth. It is not an excellent solution to save the useful resource of bandwidth [5,6]. The line code allows handling of this issue to decrease ISI, and it is also possible to use PSD at an ideal level. The different digital-to-digital line code schemes are shown in Figure 1. However, none of the single digital modulation techniques are available to meet the requirement of IoT devices to connect them with digital communication. Therefore, there is a need to design a modulation technique that provides low PSD and better utilization of bandwidth without DC components.

Existing techniques can deal with the specific nature of the problems, and with communication platforms, as with encoding techniques, some are proposed to deal with short-range and others with long-range communication. However, in the emerging field of IoT and smart technology, both types of communication are needed. The methods are used for the conversion of different kinds of signals into another form of a signal, which itself poses critical issues in communication. Because of analog-to-digital signal conversion, the data rate becomes high, and more transmission bandwidth is required for digital communication. The synchronization in digital communication is another problem that arises due to conversion methods in the case of synchronous modulation.

This work is the extension of [2]. In this paper, we propose the Shadow Encoding Scheme (SES), which modulates the signal of audio, video, and text messages into a waveform using a low PSD and without consuming high bandwidth for short- and long-range communication. SES is also able to resolve the problem of synchronization and DC components by using the shadow copy of the same repeating bits in the communication. During the programming of the SES, the digital binary string “11110000101” is used to obtain the results for comparison with other modulation schemes. The same string of bits is used for other digitizing schemes to obtain the results for bandwidth, synchronization and DC component. The string contains all possible situations in which a receiver can face difficulty to verify and check the strength of the signal.

The expected outcomes of the work are the development of a digital encoding scheme for IoT devices to communicate at a low power spectrum density. The SES enables effective communication for complex networks with lightweight signal modulation. The SES reduces unnecessary bandwidth consumption, maximizes the utilization of the power spectrum and helps to control direct current components. It has been observed from the results that SES is around 50% more effective in PSD against the other well-known digitizing schemes. SES enables lightweight communication for the IoT and sensor-based communication for smart cities.

The rest of the paper is organized as follows. Section 2 describes comprehensive related work. The SES and the details of PSD are discussed in Section 3. Section 4 presents the mathematical equations to calculate PSD. The core structure of the SES is described in Section 5. Section 6 covers the analytical assessment of the SES. Section 7 elaborates on the results and discussion. Finally, Section 8 summarizes the paper as a conclusion.

## 2. Related Work

In the literature, there has been a vast amount of research on long range (LoRa) networks. Some of the conducted research has mainly focused on comparing LoRa with other LPWA technologies such as NB-IoT and Sigfox [7]. The authors [8] explored and discussed the wide variation of performance in a specific paradigm and focused on the limitations and capabilities. The analytical and simulation-based approaches are used in these studies for experiments and performance analysis. There are many issues with the deployment of LoRa in a practical scenario. For example, LoRa does not work for digital communication: the low power promise is violated when the LoRa network transmits images, audio, video, and large size file from source to destination. Another problem that LoRa uses the radiofrequency range 300 MHz to 3 GH, which is more harmful to the human body and causes cancer. Moreover, many IoT cloud platforms have been proposed to manage and process IoT data [9].

The authors have proposed on-off keying (OOK), pulse-position modulation (PPM), digital pulse interval modulation (DPIM), pulse amplitude and position modulation (PAPM) and differential amplitude pulse modulation (DAPIM) to calculate power spectrum densities for free-space digital communication [10]. The bandwidth of these techniques has been calculated based on the occurrence of null in the PSDs. During the analysis, it was observed that the bandwidth requirement is very high in certain cases. The PSD curves are very important to design the link as these will give information on how the signal poser changes with the bandwidth. The results are helpful in the selection of modulation schemes for free-space optical links and to determine how the power content of the signals is affected by filters and other devices in the link. OOK is more spectrally efficient than frequency-shift keying but is more sensitive to noise when using a regenerative receiver or a poorly implemented superheterodyne receiver [7]. For a given data rate, the bandwidth of a BPSK (binary phase-shift keying) signal and the bandwidth of the OOK signal are equal.

The authors proposed a novel approach to realize photonic analog-to-digital conversion in a system using phase modular (PM) and delay-line interferometer (DLI) [11]. The phase modulation and interferometric demodulation techniques are used to calculate quantization and encoding. In the design system, the multiwavelength pulsed source and phase-shifted transfer function are used for signal sampling in phase modulation. Besides, the influence of amplitude and timing jitter is alleviated through a balance-detection technique. The proposed technique is not suitable when the double phase of frequency modulation occurs at the same time in the communication. In [12], the authors proposed 5G-enabled IoT gateways to communicate with remote radio heads (RRhs) of 5G C-RAN. The 5G C-RAN is endowed with compression schemes to improve uplink utilization. The experiments and results show that the C-CARN makes it possible to support a massive number of IoT devices in terms of uplink utilization through the development of a C-RAN gateway. In Centralized Radio Access Network (C-RAN), downlink communication is not considered, which is equally essential for IoT communication.

The unipolar method is a simple line-code method. It represents only one polarity format, which may be data bit 1 s or 0 s. In the case of high-polarity data bit 1 s, the waveform jumps to a positive level. The absence of waveform or low polarity represents 0 data bits. This technique is also known as ON–OFF modulation. The ON–OFF modulation technique has the drawbacks of synchronization and a DC component [12]. The unipolar method is designed for short-range communication and it is not suitable for long-range communication.

The polar method is another digital line coding method, which is further categorized into Non-Return to Zero (NRZ), Return to Zero (RZ), and bi-phase. The polar digitizing scheme uses two voltage levels, positive and negative. The DC component issue is resolved due to the usage of the negative voltage level, but the synchronization problem still exists in the polar method. NRZ uses two signal levels, positive and negative. NRZ-L is based on two voltage levels for signal representation. It helps the receiver to distinguish between positive and negative bitstreams. Synchronization still has issues in the case of 0 s and 1 s. RZ provides a good solution to transmit the 0 and 1 bitstreams over the network. The 0 s bitstream is not ignored like a previous digitizing scheme. RZ consumes double bandwidth to transmit bits over the network. Biphase is a third digitizing scheme of the polar method. Biphase is further divided into two categories, which are Manchester and Differential Manchester [13]. Manchester digitizing helps the receiver to understand the bit pattern. The authors presented power spectral density (PSD) for the nonlinear self-interference signal in a theoretical way, and the estimation method of PSD was developed for nonlinear self-interference [14]. Furthermore, the convergence performance of the canceller was improved by the proposed selection technique. The results show that the proposed method can achieve similar cancellation performance compared with the original frequency-domain Hammerstein canceller and a time-domain nonlinear canceller. The proposed approach improves the computational cost, but it requires a high-speed communication link. Furthermore, the DC component problem is not there. Double bandwidth is required in case the signals are toggled from 0 s to 1 s or vice versa [15,16]. Signal variation is decided in the middle of a transmission. The Differential Manchester digitizing scheme deals with synchronization issues in a better way. However, it requires a double bandwidth to transmit toggle bits. On the other hand, the high performance of the receiver is required in case of the same long streams. The rapid variation does not determine which bit is being transmitted over the network [17,18].

**Figure 2:**
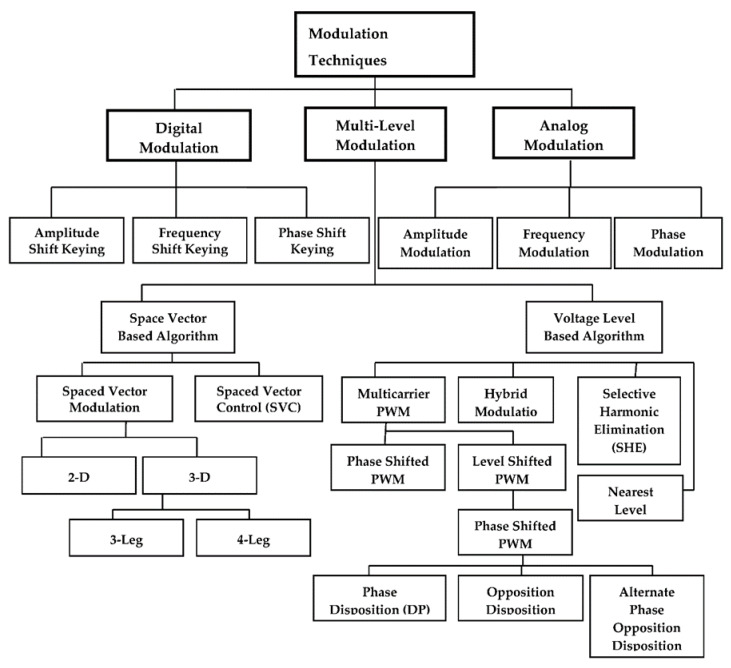
Hierarchy of the existing modulation techniques.

The bipolar method is another coding scheme which is further divided into three main subcategories: Alternate Mark Inversion (AMI), Binary 8 Zero Suppress (B8ZS), and High-Density Bipolar Order 3 (HDB3). Bipolar provides a satisfactory solution to the problems of synchronization, DC, and double bandwidth concerning other digitizing schemes. The AMI technique offers a better solution than all previously discussed digitizing schemes. In a long stream of 1 s, the receiver can quickly determine each bit. It provides a negative to positive jump in the case of 1 s bit signal modulation [19]. There is no DC component issue; no double bandwidth is required to transmit signals. Zero voltages are used to represent bit 0 s. On the other hand, it does not guarantee that the receiver will correctly receive the signal [20]. There are many issues interlinked with digital and analog signal modulation, like bandwidth utilization, the power spectrum, the short and long range of the signal, signal connectivity, etc. Therefore, several modulation techniques have been designed to resolve these issues in the domain of digital modulation, analog modulation, and multi-level modulation for network communication. Table 1 shows the related work summary against the benchmark techniques, whereas, Figure 2 presents the hierarchy of the possible existing modulation techniques for network communication.

## 3. Shadow Encoding Scheme for Power Spectrum Density

### 3.1. Power Spectral Density (PSD)

PSD is an important feature for showing the strength of frequency variations. PSD makes it easier to know which change has more and which has less power. The power of frequency is computed as per the width of obtained energy from the specific waveform unit. The waveform of a line coder shows the PSD behavior of periodic digital data. Equation (1) shows how to calculate PSD.

(1) $$P_{s}\left(f\right)=\frac{|F\left(f\right){|}^{2}}{T_{s}}\overset{\infty }{\underset{-\infty }{\sum }}R{\left(k\right)}_{e}{}_{i}^{J2\pi KT_{s}}\;$$

F(f) = Fourier transform of the pulse f(t) and R(k)

$T_{s}$ = digital binary representation time symbol $T_{s}$=$\;T_{b}$

### 3.2. Bandwidth Efficiency

Bandwidth is a useful resource for network communication. The proper use of bandwidth can increase the throughput of transmission. Bandwidth is a critical asset to evaluate the performance of network communication. The network communication system transmits the chain of bits from a communication device to the entire network. Some line codes use extra bandwidth to send data through the channel. RZ and Manchester coding schemes require double bandwidth to transmit a bit over the network [21]. SES does not require extra or double bandwidth to transmit digital bits. SES needs one $T_{b}$ for each bit of transmission.

### 3.3. Transparency

The modulated form of the digital bits into the signal at the receiver end is called transparency. Transparency makes it possible that a waveform should not affect the accuracy of the message at the destination. The long stream of 0 s and 1 s causes an error due to the stable state of the signal in a unipolar and AMI digitizing scheme. The transmission of the signal may modulate such a pattern; it should deliver at the destination faithfully. Each digital bit should represent a specific waveform for signal modulation. The proposed SES provides good transparency. The 1 bit represents the positive amplitude voltage level, and the 0 bits are modulated on a negative level [22].

### 3.4. Self-Synchronization

The sender and receiver should follow the same clock interval to make the transmission useful. If the sender’s clock is slower or faster than the receiver’s, the receiver might not be able to receive the bits. The pattern of bits would change, and 0 s may be received as 1 s at the receiver’s end or vice versa [23].

## 4. Mathematical Calculation of PSD

We have calculated the PSD for different line code techniques where the long stream of 0 s and 1 s is transmitted towards the receiver in the form of a signal. The various line codes use different energy levels for each bit of transmission. Each line code has a different waveform to represent the digital signal. The following are details of various line codes with the PSD formula.

### 4.1. Unipolar PSD

The unipolar method expresses the modulation in one signal with a width $T_{b}$. Equation (2) shows the PSD of the unipolar method in simplified form. The high polarity duration for each bit is $T_{b}$ with amplitude “A”. The low polarity or absence of a pulse “0” demonstrates the $T_{b}$. The methods of both unipolar and polar NRZ are calculated as per Equation (2) with signal plus and a DC term. Therefore, the PSD of both encoding methods is similar without the delta function in DC. “A” is used for $\sqrt{2}$, which can normalize the unity of the signal for PSD [24].

(2) $$P\left(f\right)=\frac{A^{2}T_{b}}{4}{\left(\frac{sin\pi fT_{b}}{\pi fT_{b}}\right)}^{2}\left[1+\frac{1}{T_{b}}\delta \left(f\right)\right]$$

### 4.2. Polar NRZ-L PSD

NRZ-L represents a bit with the single waveform in $T_{b}$, the plus duration for polar NRZ-L of PSD. Equation (3) shows the PSD of NRZ-L. In Equation (3) “A” represents the 1 bit, which normalizes the unity average of PSD signal. NRZ-L requires a double voltage level for illustration of the 0 s and 1 s in the bitstream [25].

(3) $$\left(f\right)=A^{2}T_{b}{\left(\frac{sin\pi fT_{b}}{\pi fT_{b}}\right)}^{2}\;$$

### 4.3. Bipolar RZ (RZ-AMI) Signals PSD

Return to Zero (RZ) is based on positive and negative voltage levels. The “1” is illustrated with “A”, positive amplitude, while a “0” is mapped with “−A”, negative amplitude. In some cases, bipolar RZ and AMI are considered with the same amplitudes. The amplitudes $(a_{k+nk}$ in both instances use different levels of voltage (+A, 0, −A). The “1” is modulated amplitude, “A” and “–A”, alternately, while the “0” corresponds to zero voltage as shown in Equation (4) [26].

(4) $$P\left(f\right)=\frac{A^{2}T_{b}}{4}{\left(\frac{sin\pi fT_{\frac{b}{2}}}{\pi fT_{\frac{b}{2}}}\right)}^{2}{sin}^{2}(\pi fT)\;$$

### 4.4. Manchester PSD

Manchester digital format uses double bandwidth in signal modulation, but there is no DC level in Manchester signal propagation. Equation (5) shows that the PSD formulation of the Manchester coding scheme has good results due to its zero power at zero frequency [27].

(5) $$P\left(f\right)=\frac{A^{2}T_{b}}{4}{\left(\frac{sin\pi fT_{\frac{b}{2}}}{\pi fT_{\frac{b}{2}}}\right)}^{2}{sin}^{2}(\pi fT_{\frac{b}{2}})$$

## 5. Core Structure of Ses Transmission

**Figure 3:**
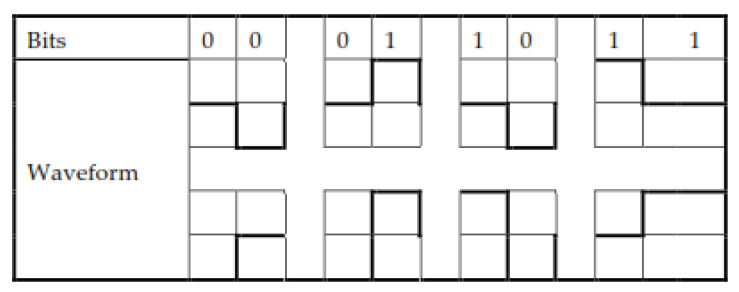
The basic structure of SES.

SES is based on three voltage levels (+A, 0, −A). Therefore, SES belongs to a bipolar format to represent the binary digits. SES presents bit 1 with a high voltage amplitude, +A. In the case of consecutive 1 s, SES represents the second 1 with a level 0 voltage. The 0 is represented in SES as a “−A” waveform amplitude; in the case of repeating 0 s, the second 0 bit is shifted over the 0 voltage level. The logic 0 has a high probability because it represents both logics in consecutive cases. The basic signal modulation pattern against digital bits (0, 1) and the mapping of the digital bitstream (11110000101) modulation in SES into the waveform is shown in Figure 3.

**Figure 4:**
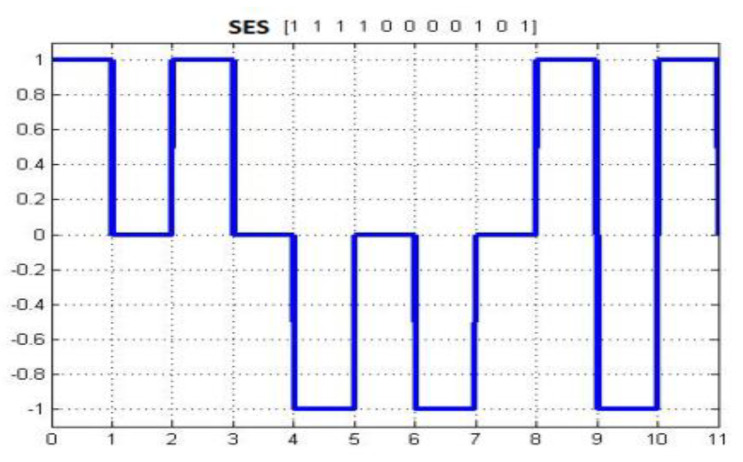
Shadow Encoding Scheme (SES) waveform.

The basic structure of signal modulation in SES is shown in Figure 4. It has been observed that there is no synchronization and double bandwidth usage for bit representation in the waveform.

### 5.1. Comparative Analysis of SES with Other Digitizing Schemes

**Figure 5:**
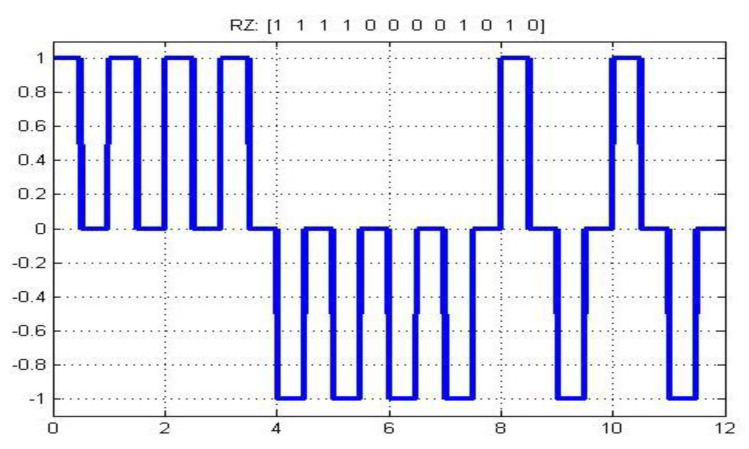
RZ waveform.

**Figure 6:**
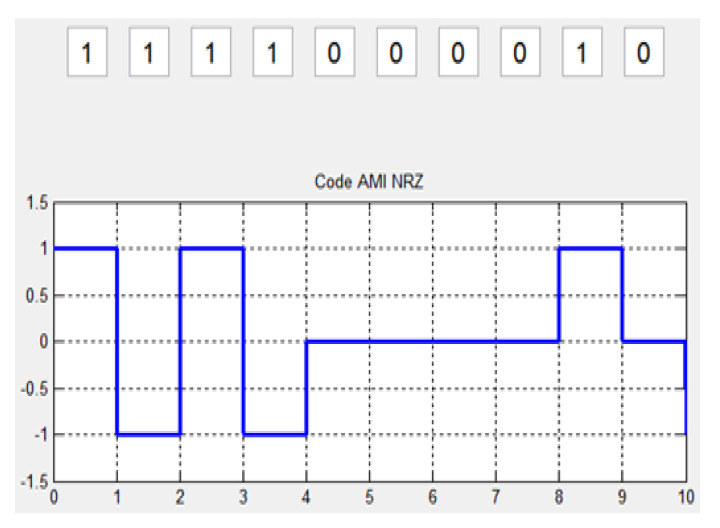
SES AMI.

SES has some similarities with other digital encoding schemes. These similarities improve the reliability and validity of SES. Two main similarities exist in SES, with the digitizing scheme RZ shown in Figure 5 and AMI has shown in Figure 6.

The SES level returns to zero after modulating a signal. The same modulation is performed in RZ. The difference is that RZ uses double bandwidth, and SES does not require this.

AMI jumps from −A to +A and vice versa on a long stream of 1 bit. The SES signal variation is from +A to −A or vice versa when the string goes from 1 to 0 or 0 to 1.

### 5.2. Autocorrelation in Function Data

Autocorrelation points to the matching of the process signals with the delay of simplification. The method of the autocorrelation function of data R(k) is defined in Equation (6) [28].

(6) $$R\left(k\right)=\overset{i}{\underset{i=1}{\sum }}(a_{n},a_{n+k})p_{i}$$

$a_{n}$: The first value, which is 0 or 1, of the digital bits (0,1)

$a_{n+k}$: The ${\left(n+k\right)}^{th}$ Tb value, which has a kth position of bits (00, 01, 10, 11)

$p_{i}$: The probability of accruing the digital bit in the i^th^ symbol position $\;(a_{n+kn}$ product.

### 5.3. Line Coder Equation of Physical Waveform

The line encoder follows the specific pattern of value for $a_{k}$. It is the function of digital input bits or an ADC output. Equation (7) is used for the encoder output waveform.

(7) $$x\left(t\right)=\overset{\infty }{\underset{k=-\infty }{\sum }}a_{k}p\left(t-kT_{b}\right)$$

p(t): The waveform input data bits according to the line code scheme.

$T_{b}$: The position for calculating the digital bit period.

$R_{b}$: $\frac{1}{T_{b}}=nfs$, for i^th^ bit iteration.

**Figure 7:**
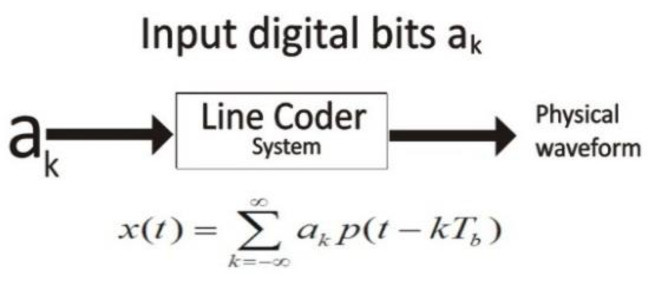
Line Coder System.

The line coder takes as input $a_{k}$ digital bits and converts them into a physical waveform, according to the modulation of the adapted line coding scheme. Figure 7 shows how the line coder system transfer digital bits into a physical waveform [29,30].

## 6. Analytical Assessment of SES

SES uses an exceptional method to represent a bit in three possible signal states. The amplitude $\left(a_{k},a_{k+n}\right)$ may be used for 1 s, having “A“ amplitude and 0 levels, while a logic 0 can illustrate the signal level “–A“ and 0. The low state of 0 is used in both cases of logic (0, 1). The probability of 0 is ½ and the probability of +A and –A is ¼. Therefore, SES uses a different adjacent bit pattern with various past line codes. It is due to the symmetric level of SES, which affects the behavior of autocorrelation. The possibilities of digital bit transmission are equal to ½. PSD is based on two main factors: one is the pulse shape (line coder) and the second is autocorrelation [31]. Equation (6) shows the simplified form of autocorrelation, and Equation (7) represents the physical waveform of line encoding. The simplified form of the PSD digital signal is shown in Equation (1).

There are three possible levels of signal representation in SES. The signal demonstration has the specific levels +A for 1 and −A for 0, and 0 symbols are used for both 1 s and 0 s for a second consecutive bit with a probability of ½. Table 2 shows the probabilities of inputs of digital bits and a description of the bits with their likelihood.

There are different possibilities for the digital bits occurring in a data stream. The data stream may have 0 at the start of the stream or 1. With the next bit, the possibilities increase to 4, such as 00, 01, 10, or 11. Applying this method, three-bit options extend up to 8 possibilities, and so on. This autocorrelation behavior extends in an exponential form, which is difficult to calculate. The autocorrelation for n = 0 is shown in Equation (8).

For n = 0

(8) $$\begin{array}{l} R0=A.A\frac{1}{4}+\left(-A.-A\frac{1}{4}\right)+0.0\frac{1}{2}\\ =\frac{A^{2}}{4}+\frac{A^{2}}{4}+0\\ =\frac{2A^{2}}{4}=\frac{A^{2}}{2} \end{array}$$

Table 3 shows the case of n = 1 with consecutive adjacent bits. The autocorrelation necessitates the difference for |n| = 1.

Next is the last case of |n| > 1, which has a possible combination for n > 1.

The combinations of bits in three-bit logic are (000, 001, 010 … 111). Now, all of these bits have equal probability. $\left(a_{k},\;a_{k+n}\right)$ = 0.

Equation (9) shows all the results of the autocorrelation $R_{A}\left(n\right)$. The autocorrelation results are calculated for n = 0, n = 1, and n > 1.

(9) $$R_{A}\left(n\right)=\left[\begin{array}{l} \frac{a^{2}}{2},n=0\\ \frac{-a^{2}}{4},n=+A\\ \frac{a^{2}}{4},n=-A\\ 0,n>1 \end{array}\right]\;$$

Equation (10) shows the general format of PSD with autocorrelation and line code. The PSD is formulated for SES after applying autocorrelation and probability factors.

(10) $$\begin{array}{l} S_{x}\left(f\right)=\frac{1}{T_{b}}\left[T_{b}{}^{2}{\left(\frac{sin\pi fT_{b}}{\pi fT_{b}}\right)}^{2}\right]\left[R_{A}\left(-1\right)e^{j2\pi fbTb}\right]+R_{A}\left(0\right)+\left[R_{A}\left(1\right)e^{-j2\pi fbTb}\right]+R_{A}\left(0\right)\\ S_{x}\left(f\right)=\left[T_{b}{\left(\frac{sin\pi fT_{b}}{\pi fT_{b}}\right)}^{2}\right]\left[\frac{a^{2}}{2}-\frac{a^{2}}{4}\left(e^{j2\pi fbTb}+e^{-j2\pi fbTb}\right)\right]\\ S_{x}\left(f\right)=\left[\frac{a^{2}T_{b}}{2}{\left(\frac{sin\pi fT_{b}}{\pi fT_{b}}\right)}^{2}\right]\left[1-\cos(2\pi fbTb)\right]\\ S_{x}\left(f\right)=\left[\frac{a^{2}T_{b}}{2}{\left(\frac{sin\pi fT_{b}}{\pi fT_{b}}\right)}^{2}\right]\left[2{sin}^{2}(\pi fT_{b})\right]\\ S_{x}\left(f\right)=\left[a^{2}T_{b}{\left(\frac{sin\pi fT_{b}}{\pi fT_{b}}\right)}^{2}\right]\left[{sin}^{2}(\pi fT_{b})\right] \end{array}$$

Equation (4) shows that each $T_{b}$ requires double bandwidth in bipolar RZ and AMI. SES has similarities of signal modulation with RZ and AMI, except for dual bandwidth usage. SES does not include $T_{b/2}$, so the equation of SES for PSD is shown in Equation (11).

(11) $$P_{SES}\left(f\right)=\frac{A^{2}T_{b}}{4}{\left(\frac{sin\pi fT_{b}}{\pi fT_{b}}\right)}^{2}{sin}^{2}(\pi fT)$$

## 7. Results and Comparisons

**Figure 8:**
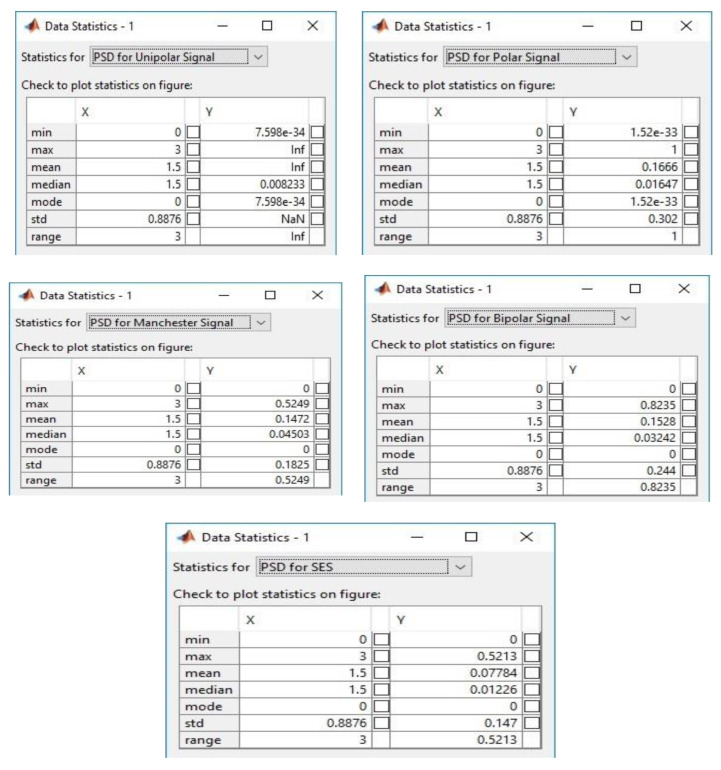
Statistical comparison of SES with other digital encoding schemes.

The simulation of SES was done in MATLAB (Mathworks Inc., Natick, MA, USA) to compare the performance of modulation, in terms of PSD and bandwidth utilization, between the unipolar, polar, bipolar, and Manchester methods. Firstly, the coordinates of “x” and “y” were measured using the statistical method to analyze the performance of various line codes as shown in Table 4. The principal of the statistical model is based on mean, mode, and standard deviation to validate the results. The statistical analysis of PSD, which has been performed for SES and other well-known modulation techniques based on the properties of line codes, is given below. The statistical comparison and configuration setup are illustrated in Figure 8.

- Min = the starting minimum value.
- Max = the ending maximum value.
- Mean = average of x and y coordinates.
- Median = midpoint of the Min and Max values.
- Mode = mode of analysis values.
- STD = standard deviation.
- Range = range of plated values.

Secondly, the SES was also compared with the IoT-based modulation technique Chirp Spread Spectrum (CSS), with the configuration of the LoRA and SigFox communication network that is dedicated to IoT devices in smart cities. A total of 15 data frames were generated in the network between the sender and receiver to calculate the bit error rate (BER). The signal-to-noise ratio (SNR) is also taken in the simulation to compare SES with CSS. The SNR simulation configuration is based on Equation (12), which is taken from [32].

SNR (dB) ≈ 20 log10 A + 6.02bits + 9.03r − 3.41.(12)

**Figure 9:**
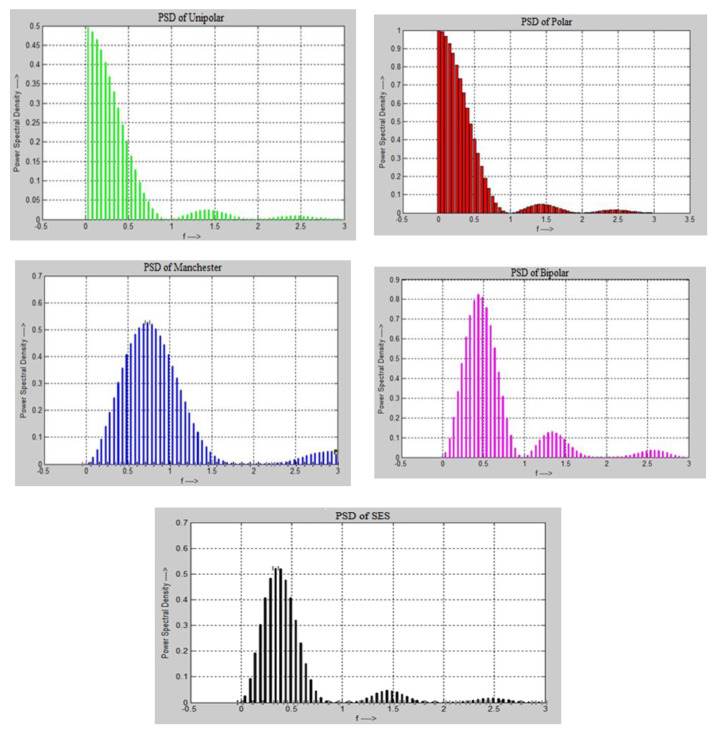
PSD calculation of SES with other digitizing schemes.

Equations (1)–(5) and (9) have been used to calculate PSD for various line codes. The graphical representation of results for multiple line codes and SES against PSD is shown in Figure 9. It seems that SES utilizes PSD better than the other digital-to-digital encoding schemes. This is because SES does not modulate the signal repeatedly, due to the forwarding of a shadow copy of the same bitstream without performing modulation.

Table 4 presents a comparison of the x and y coordinates of SES with the unipolar and polar methods. The total of the “y” column for unipolar is 4.264236, and SES’s is 4.2587352. SES almost uses the same PSD as unipolar. Unipolar is based on just an +A amplitude with the DC component, and has synchronization issues, while SES is based on different level voltages without synchronization problems. The difference between the two digitizing approaches is only 0.0144992. The difference in PSD between SES and polar is 5.2493118 to modulate a signal. The polar digitizing scheme requires almost 44% more power as compared to SES. Therefore, SES has a better performance in signal modulation.

The Manchester encoding scheme provides a good solution, but it requires double the bandwidth to transmit a bit. During the simulation of Manchester and calculation coordinates, it was determined that it needed extra energy for bits modulation. Table 5 shows the “x” and “y” coordinates of the Manchester and bipolar encoding schemes. The Manchester encoding scheme is expensive on the PSD property. The SES requires very low power in comparison to the Manchester coding scheme. The bipolar encoding scheme uses three voltage levels. At the co-ordinate values of x and y, where the total 8.89774 PSD is used by bipolar and SES uses less power spectrum as compared to bipolar. The difference between both digitizing schemes is 4.6190048. The bipolar is required 48% more power for data bits transmission. SES again achieves a better performance than the bipolar digitizing scheme.

### SES Comparison with Various Line Codes

**Figure 10:**
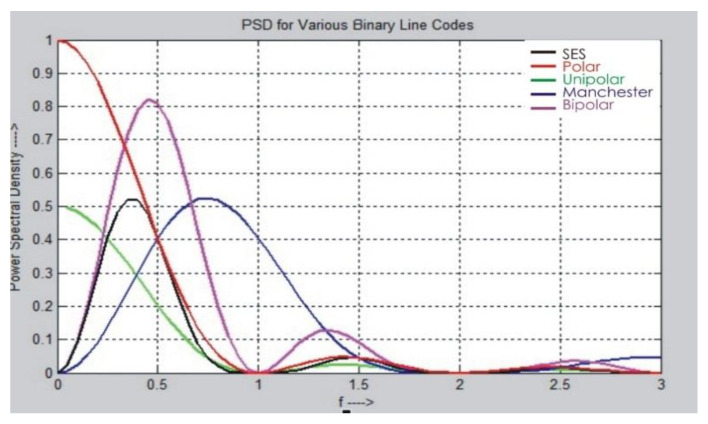
SES vs. Various line codes.

Figure 10 shows the PSD comparison of selected digitizing schemes in a line graph. It has been observed that SES used PSD better than the other digitizing schemes. SES utilizes less bandwidth because the shadow copy of the same previous bitstream is transferred, rather than the original stream of bits. The SES works in the light mode of conversion of data bits because one stream of the data bits is converted and the rest of the data stream is forwarded in the shadow form of the previous data bitstream. It is observed that, as frequency increases, the PSD utilization is reduced, and with a decrease in frequency, PSD utilization increases.

**Figure 11:**
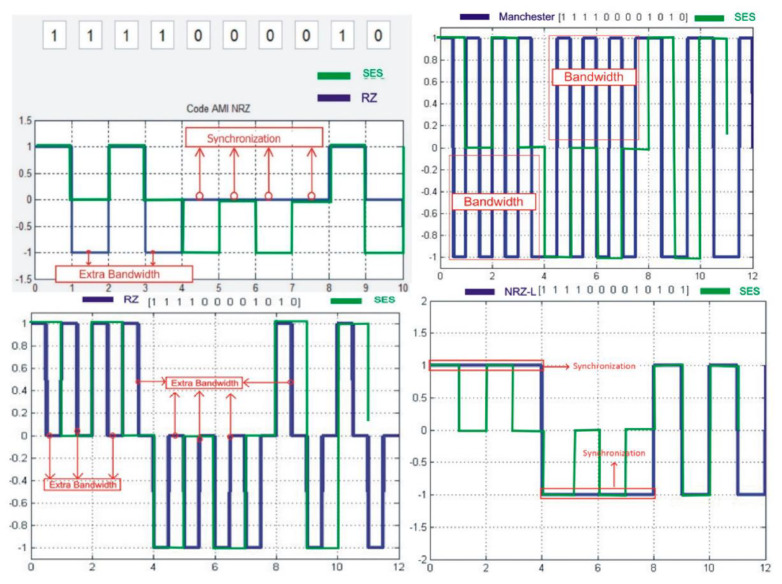
PSD and bandwidth utilization between SES and other digital encoding schemes.

Some digitizing schemes waste the most useful resource, network bandwidth while transmitting the data: for instance, RZ uses double bandwidth for each bit transmitted over the network. However, the SES uses each Tb for each bit of data. Double bandwidth is not required in the case of SES. All bits of data are sent over the network in each Tb per bit. It is shown in Figure 11 that SES performed better than other digitizing schemes in terms of bandwidth utilization and synchronization problems. The SES presents that each bit has one Tb, which is mapped for the signal. The slices of Tb help the receiver to understand the starting and ending position of the signal. Figure 11 shows that there is no tight variation in the signal, as with RZ. The strict variation in the signal is bound to the receiver, which cannot lose its timing in any case. The SES defense against the bandwidth is very descriptive and is less bound to the receiver.

**Figure 12:**
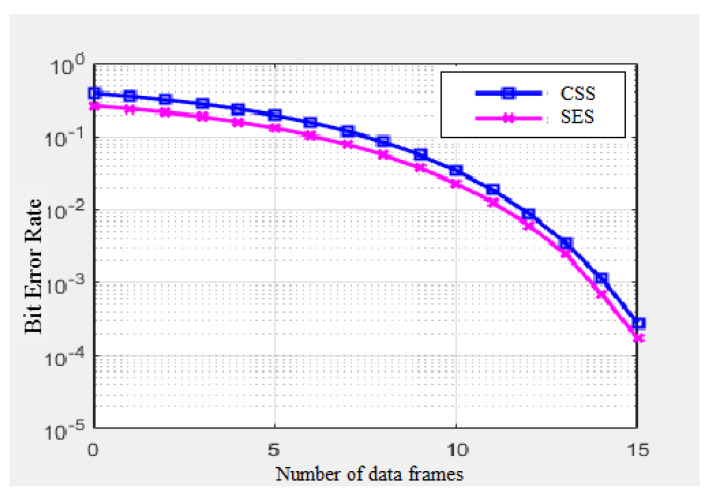
Bit error rate of SES vs. CSS.

Figure 12 presents the bit error rates between SES and CSS. It has been observed that, as the number of data frames increases in the network, the bit error rate increases. The results show that SES modulation performs better than the CSS modulation technique. The bit error rate is calculated based on audio, video, and text messages. The SES provides support to modulate three formats (audio, video, and text messages) of data, but, on the other hand, CSS is purely designed for text messages for IoT communication.

**Figure 13:**
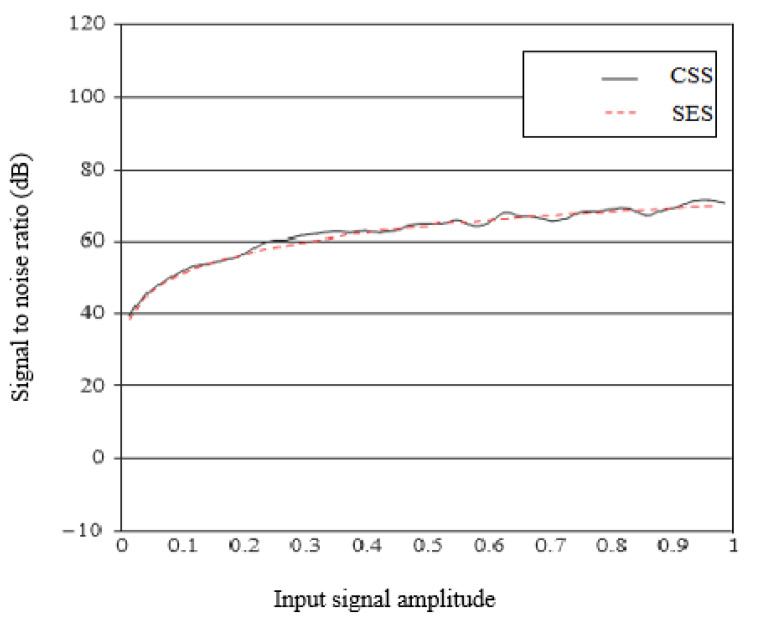
Signal to noise ratio of SES vs. CSS.

Figure 13 illustrates the difference in SNR between SES and CSS. It seems that there are few variations in SNR as the amplitude of the input signal increases. Similar modulation behaviors can be observed for SES and CSS in the calculation of SNR. When the modulators are in the stable region of operation, we observed a close match of the approximated SNR results during the simulation between SES and CSS.

## 8. Conclusions

This paper presents the proposed SES line code scheme to deal with PSD and network bandwidth for IoT-based communication. The SES is compared with other well-known line code techniques. The comparison is performed concerning statistical analysis and PSD calculation of waveforms with x and y coordinates of PSD values. The “x” coordinates show the fixed interval of a waveform, which is almost 0.5. The next column, “y”, shows the waveforms of PSD variations.

The SES is a better digitizing scheme in many respects. The SES does not require the double bandwidth to transmit digital bits over a transmission medium in signal form. The SES does not have a synchronization issue due to the toggle nature of the signal. The SES looks better for fixing the DC component issue due to the use of three voltage levels for signal presentation. The SES provides a better solution for the BER and SNR, concerning CSS modulation for smart cities. SES uses the ideal power to transmit a signal over the channel. The results for PSD are 50% better than common digitizing schemes. SES is also applicable to IoT-based communication in smart cities’ ecosystems. To improve the computation cost and speed of signal modulation for short and long-distance communication, as well as the implementation of code on real hardware such as the ESP32 or STM32 (B-L072Z-LRWAN1) platforms, can be the future direction of this research work.

## Figures and Tables

**Table 1:** Related work summary against the benchmark techniques.

Encoding Techniques	Features	Applications	Achievements	Limitations
Chirp Spread Spectrum (CSS) [7]	IoT communication	Customized application of MDMA for the requirements of battery power	Enables IoT communication on a large scale	Uses the radiofrequency range 300 MHz to 3 GH, which is more harmful to the human body and causes cancer
Unipolar [12]	Digital to a digital encoding scheme	Digital short-range systems	It requires low bandwidth	Synchronization is required and DC components exist
Non-return-to-zero level (NRZ-L) [13]	Digital to a digital encoding scheme	Wired Lan	Uses low bandwidth No DC component	Lack of synchronization for the long bitstream
Non-return-to-zero inverted (NRZ-I) [13]	Digital to a digital encoding scheme	Wired Lan	Uses low bandwidth No DC component	A systemic problem with polar is that the polarity can be backward
Non-Return to Zero (NRZ) [14]	Digital to a digital encoding scheme	Wired Lan-RS232-based protocols	It is simpler and requires low bandwidth	No error correction was done. The presence of low-frequency components may cause signal droop. No clock is present. Loss of synchronization is likely to occur (especially for long strings of 1 s and 0 s).
Return to Zero (RZ) [16]	Digital to digital encoding scheme	Wired Lan	It is simple. No low-frequency components are present.	No clock presents No error correction Occupies twice the bandwidth of NRZ
Manchester [18]	Digital to a digital encoding scheme	Used in 802.5 (token ring) with twisted pair	No DC component Easy to synchronize	Modulation rate for Manchester is twice the data rate Inefficient encoding for long-distance applications
Differential Manchester [20]	Digital to a digital encoding scheme	Used in 802.5 (token ring) with twisted pair	No DC component Transparent Does not suffer from signal droop	Required high bandwidth for the long stream of data bits Inefficient encoding for long-distance applications
Alternate Mark Inversion (AMI) [21]	Digital to a digital encoding scheme	This technique is suitable for transmission over AC coupled lines, as signal drooping does not occur here.	It is simple. No low-frequency components are present. It occupies lower bandwidth than unipolar and polar NRZ schemes. A single error detection capability is present in this.	No clock is present The long string of data causes loss of synchronization

**Table 2:** Digital bit probabilities in SES.

Input	Probability	Description
P(Ak=+a)	$\frac{1}{4}$	It is for bit 1
P(Ak=−a)	$\frac{1}{4}$	It is for bit 0
P(Ak=0)	$\frac{1}{2}$	It is for second consecutive bit 0 or 1

**Table 3:** Autocorrelation values for n = 1.

Adjacent Bits	(0, 0)	(0, 1)	(1, 0)	(1, 1)
$\left(a_{k},a_{k+n}\right)$	−A, −A 0, 0	−A, 0 0, +A	+A, 0 0, −A	+A, +A 0, 0
	(−A2)	0	0	(A2)

**Table 4:** SES vs. Unipolar and Polar coordinates.

SES Signal		Unipolar Signal		Polar Signal	
x	y	X	y	x	y
0	0	0.05	0.4959	0	1
0.05	0.02427	0.1	0.4838	0.05	0.9918
0.1	0.09239	0.15	0.4641	0.1	0.9675
0.15	0.1913	0.2	0.4376	0.15	0.9281
0.2	0.3024	0.25	0.4053	0.2	0.8751
0.25	0.4053	0.3	0.3684	0.25	0.8106
0.3	0.4823	0.35	0.3283	0.3	0.7368
0.35	0.5213	0.4	0.2864	0.35	0.6566
0.4	0.5181	0.45	0.2441	0.4	0.5728
0.45	0.4762	0.5	0.2026	0.45	0.4881
0.5	0.4053	0.55	0.1634	0.5	0.4053
0.55	0.3188	0.6	0.1273	0.55	0.3267
0.6	0.2303	0.65	0.09519	0.6	0.2546
0.65	0.1511	0.7	0.06767	0.65	0.1904
0.7	0.08858	0.75	0.04503	0.7	0.1353
0.75	0.04503	0.8	0.02735	0.75	0.09006
0.8	0.0189	0.85	0.01445	0.8	0.0547
0.85	0.005957	0.9	0.005972	0.85	0.0289
0.9	0.001141	0.95	0.001374	0.9	0.01194
Total	4.2787352	Total	4.264236	Total	9.528047

**Table 5:** SES vs. Manchester and Bipolar coordinates.

SES Signal		Manchester Signal		Bipolar Signal	
X	y	x	y	x	y
0	0	0	0	0	0
0.05	0.02427	0.05	0.006143	0.05	0.02442
0.1	0.09239	0.1	0.02427	0.1	0.09471
0.15	0.1913	0.15	0.0535	0.15	0.2023
0.2	0.3024	0.2	0.09239	0.2	0.3343
0.25	0.4053	0.25	0.1391	0.25	0.4748
0.3	0.4823	0.3	0.1913	0.3	0.6075
0.35	0.5213	0.35	0.2466	0.35	0.7171
0.4	0.5181	0.4	0.3024	0.4	0.7916
0.45	0.4762	0.45	0.3561	0.45	0.8235
0.5	0.4053	0.5	0.4053	0.5	0.8106
0.55	0.3188	0.55	0.4479	0.55	0.7557
0.6	0.2303	0.6	0.4823	0.6	0.6665
0.65	0.1511	0.65	0.507	0.65	0.5536
0.7	0.08858	1	0.4053	0.7	0.4298
0.75	0.04503	1.05	0.3631	0.75	0.3075
0.8	0.0189	1.1	0.3188	0.8	0.1979
Total	4.2787352	Total	4.34	Total	8.89774
